# Understanding health and social care

**Published:** 2013-05-31

**Authors:** Przemyslaw M Sowa

**Affiliations:** Australian Centre for Economic Research on Health, The Australian National University, Australia. Email: prem.sowa@anu.edu.au

Conventional thinking conveniently conceptualises health and social care as self-contained areas, with their respective spheres of financing, provision and research. Jon Glasby challenges this over simplification by demonstrating a continuum of services that has problematic implications for inter-agency coordination and distribution of responsibility.

In setting the stage, “Understanding Health and Social Care” tracks the history of the welfare sector back to its charity and voluntary action roots in the early 19th century. From that point onwards, it tells the story of the gradual formalisation of both social services and the National Health Service, accompanied by an evolution of practices, emergence of health professions and developments in social thought. The opening chapters throw light on the historical dependency of the existing structures, and offer an appraisal of the current system’s capacity to respond to needs of the elderly, people with mental health problems, chronic conditions, learning difficulties, and those qualifying to multiple aforementioned groups.

The remaining chapters explore five knotty areas of tangency, beginning with the nature and challenges of partnership. Differences between health and social care encompass administrative and geographical decentralisation, financing and regulatory bodies, eligibility criteria, user charges, and above all the underlying philosophy of care. An example of faulty care continuity is the NHS-conducted hospital discharge, where lack of planning, poor communication, and inadequate support for carers persist as sources of dissatisfaction and inefficiency.

Further, the value of independent living is highlighted. Glasby explains that ‘disabled’ and ‘dependent’ are too often taken for synonyms, leading to patients’ disempowerment. Based on this observation he argues for a broader social interpretation of care that does not cease at medical treatment, but aims to create conditions of personal choice, control and freedom. In considering factors that constrain life opportunities, this extended definition places social barriers on an equal footing with impairment due to a medical condition. The personal stories invoked in the chapter conduce to a humane understanding of the problem, and send a powerful message that independence is the indispensable means for dignity and attainment of individual aspirations.

Despite the long-standing commitment to equality in British welfare, various individual and institutionalised forms of discrimination have persisted. Given the lack of systemic measures aimed at closing the existing gaps, application of the “equal treatment for equal need” principle can at best perpetuate the inequalities. The discussion of equitable care extends to the topic of patient empowerment, which, despite being well-grounded in consumer, citizen and human rights, continues to be seen as ‘optional’. Glasby presents numerous forms of user involvement, with particularly engrossing illustrations of how seemingly pro-consumer actions such as representation and opinion polling can be turned around to reinforce existing practices that put medical professionals in the position of power.

The closing chapter is devoted to “health’s forgotten partners”. Predominantly under the radar, informal carers provide a wide array of services of great materiality. There are six million carers in the UK alone, whose dedication reduces the pressure on the public system by an estimated £87 billion. This massive effort, coupled with considerable expertise, ought to position them centrally in the system. In reality, they often report lack of appreciation and exclusion from decision-making by professionals who to a much smaller extent bear the burden of caring. In addition, carers are exposed to distinctive sources of stress and health risks, as well as inequalities of workload, and thus require financial and non-financial support. Currently, those needs are only met in part.

The composition of Glasby’s book corresponds well to the goal of providing a comprehensive and up-to-date analysis of adult social care and community health. A series of readable chapters reveal some less tangible aspects of the welfare sector organisation. A lasting impression is that the author values the readers’ time by providing key messages in a clear and succinct manner. For the most part, the level of generality is adequate enough to make the findings relevant to countries other than the UK.

International readers may find the British political context over-emphasised, however. The narrative frequently refers to party politics, without making clear the extent to which declarations materialise in the form of new and existing institutions. The impression of British-centredness is deepened by curt notes on other countries’ practices, which are signalled rather than presented. The book could provide figures more generously to show the weight of the discussed problems and illustrate the advocated programmes with evidence of cost-effectiveness valuations. Whereas focusing on the discourse may leave some readers speculative about the economic and medical aspects.

This is not necessarily an issue, however, because Glasby’s interest and understanding of health and social care is openly rooted in cultural, historical and political processes that shape the NHS and other welfare agencies. Much attention is given to the evolution of socio-political concepts (e.g. ‘big society’), slogans (“we are all in this together”) as well as to positive and negative loadings of terms that determine the sector rhetoric (informal care—inferior care?).

To be sure, the book offers valuable insights for integrated care professionals, for two reasons. Firstly, the broad environmental understanding of health and social needs implies that integrated care should account for community services complementarily to medical care. Secondly, similar obstacles are likely to appear in any partnership that brings together different organisational cultures and professional philosophies. Continuity of integrated care hinges not only on bearing the cost of service, by means of in-kind provision or direct payments, but also on effective inter-agency coordination of responsibility, information-sharing and decision-making. Historically, distribution of these assets has been biased towards the NHS.

The second edition of the book arrives at the painful time of public sector austerity. Smarter caring is no longer seen as a possibility, but a necessity. Glasby’s concise manuscript will make anyone involved in streamlining health care aware of the pitfalls of joined-up services. Between information technology advancements, the welfare sector downscaling, and the fact that already a quarter of UK adults are eligible for health or social care services, the inter-agency models of provision are only bound to increase in materiality.

